# Macrophages and Neutrophils: Regulation of the Inflammatory Microenvironment in Autoimmunity and Cancer

**DOI:** 10.1155/2016/5894347

**Published:** 2016-09-20

**Authors:** Michal A. Rahat, Seth B. Coffelt, Zvi Granot, Munitta Muthana, Amedeo Amedei

**Affiliations:** ^1^Carmel Medical Center and The Ruth and Bruce Rappaport Faculty of Medicine, Technion, 3436212 Haifa, Israel; ^2^CRUK Beatson Institute, Institute of Cancer Sciences, University of Glasgow, Glasgow G61 1BD, UK; ^3^Department of Developmental Biology and Cancer Research, The Institute for Medical Research Israel-Canada, The Hebrew University-Hadassah Medical School, 91120 Jerusalem, Israel; ^4^Academic Unit of Inflammation & Tumor Targeting, Department of Oncology & Metabolism, The Medical School, University of Sheffield, Sheffield S10 2RX, UK; ^5^Department of Experimental and Clinical Medicine, University of Florence, 350134 Florence, Italy

Macrophages and neutrophils are phagocytes that play major roles in the onset and maintenance of many diseases. These two cell types that belong to the innate immune system are extremely plastic and can move between different modes of activation upon cues received from their immediate microenvironment [1–3]. Once activated, these cells secrete myriad of mediators that shape and regulate the microenvironment, as well as other immune cells, such that this continuous dialogue determines the direction of the immune response and its outcome [4]. This is highlighted in this issue as we focus on the role of macrophages and neutrophils in both cancer and autoimmune diseases. Although these are different diseases, with opposing pathophysiologies and activation of the immune system, some similarities do exist [5]. By comparing these two cell populations in cancer and autoimmune diseases, in the context of their respective microenvironment, we try to examine whether there are similar attributes that could potentially be exploited as new therapeutic strategies. Most of the manuscripts in this issue are dedicated to cancer and the tumor microenvironment (TME), reflecting the abundance of information on macrophages, and the now growing recognition of the role that neutrophils play in the cancerous context. In contrast, the role that both macrophage and neutrophils play in autoimmune diseases is only beginning to emerge and merits more investigation.

We begin with the remote microenvironment. As the TME can remotely affect circulating blood cells, O. Balacescu et al. performed transcriptional analysis in blood samples derived from triple negative (HER2-ve) breast cancer patients (TNBC). These studies revealed distinct molecular signatures according to estrogen and progesterone receptor (ER and PR) status. They found significant enrichment of altered systemic immune-related pathways in the blood of TNBC patients and this correlated with an increase in inflammation and necrosis in primary tumors. The authors also propose that immunotherapy could possibly be synergistic to chemotherapy to improve the clinical outcome of these patients.

In a series of papers, the role of macrophages in cancer is addressed, as well as their potential to become targets or vehicles of therapy. C. Eyileten et al. highlight the role of immune cells and the cellular factors they produce, in promoting or preventing cancer development. They describe how these cells, which include tumor-associated macrophages (TAMs), dendritic cells, neutrophils, T cells, and NK cells, can be targeted or exploited to induce antitumor immunity and discuss the pioneering studies where these cells have been manipulated to exhibit antitumor activity.

The ability of TAMs to produce IL-6 and activate STAT3 has been associated with chemoresistance and poor prognosis in several cancers including colorectal carcinoma. However, it remains unclear whether anticytokine therapy could reverse this, help regain chemosensitivity, and enhance the suppressive effect of chemotherapy on tumor growth. Z.-Y. Wang et al. demonstrate that treatment of carboplatin increased IL-6 production and STAT3 activation in a dose-dependent manner in the human colorectal LoVo cells, whereas anti-IL-6 neutralizing antibody enhanced their chemosensitivity to carboplatin, abolished STAT3 activation, and increased cell apoptosis. These results suggest a new way to increase the efficacy of chemotherapy.

Nonresolving inflammation is one of the consistent features of the tumor microenvironment and in the intestine; this plays a critical role in the initiation and development of colon cancer. In the study by Y. Wang et al., the inhibitory effect of a novel soy-protein-derived isoflavonoid on inflammation-related colon cancer cell proliferation is described. The anticancer role of a newly synthesized derivative of genistein, GEN-27, was shown to have both antiproliferative and anti-inflammatory properties in colon cancer cells and monocytic cells via modulation of the NF-*κ*B pathway.

N. J. Brady et al. discuss macrophages in the context of normal mammary gland development and mammary tumorigenesis. The review highlights the vital role of macrophages in mammary gland generation and homeostasis, as well as their contribution to tumor formation and progression.

B. Sainz Jr. et al. review the current literature on cancer stem cells and macrophages. This article focuses on the molecular crosstalk between the two cell types within the premalignant niche and established tumors, which influences cancer progression.

We then proceed with the introduction of neutrophils and their function in cancer, as well as their interactions with macrophages. Z. Granot and J. Jablonska review the pro- and antitumor properties neutrophils exhibit, which are regulated by cues in the tumor microenvironment. Much like macrophages, neutrophils are not a homogeneous population of cells and can become either protumoral (N1) or antitumoral (N2). Moreover, neutrophils have a major role in generating the premetastatic niche, as indicated by the large number of neutrophils accumulated in such sites. However, whether neutrophils are activated as N1 or N2 is dictated by the TME. Much remains unknown about the possible activation modes of neutrophils, their biological markers, and their functions in the polarized state in the tumoral context.

M. Orozco-Morales et al. review the interplay between molecular inflammatory mediators and the immune cells recruited to Non-Small Cell Lung Cancer (NSCLC). They highlight the roles played by various factors in regulating the function of TAMs and Tumor-Associated Neutrophils (TANs) in the context of NSCLC. Finally, they discuss the role of tumor cell expressed CD47 in mediating immune evasion.

The inflammatory microenvironment, as studied especially in tumors but also in inflammatory diseases such as rheumatoid arthritis (RA), is very hypoxic, and the lack of available oxygen influences both macrophages and neutrophils, as discussed by A. Egners et al.. Hypoxia induces or activates major transcription factors, such as hypoxia inducible factors (HIFs) 1 and 2 and NF-*κ*B, and those in turn mediate and regulate the hypoxic stress. This is manifested by major changes in every aspect of macrophage and neutrophil functions, including their migration and adhesion to endothelial cells, their ability to kill bacteria, their metabolism and polarization, production of cytokines, and protumorigenic activity, as reviewed in this paper.

The tumor microenvironment (TME) polarizes TAMs and TANs to become protumoral and support tumor growth and progression, invasiveness and metastasis, angiogenesis, and matrix remodeling, while inhibiting the antitumoral immune surveillance. In inflammatory microenvironments and in TME, neutrophils can recruit macrophages, and these, in turn, affect neutrophil functions, thereby exhibiting different degrees of interaction between these two cell types. Kim and Bae explore the biology of TAMs and TANs and their recruitment and polarization and discuss their possible interactions in the TME as well as their role in TME maintenance and their significance in clinical settings. They concluded that the introduction of more sophisticated tumor models and techniques to differentiate different myeloid cell subsets* in vivo* will reveal fundamental information about possible modulation of myeloid cells, including their interaction with platelets in each progression stage of different cancer types.

To finalize the issue, we address the role of macrophages and neutrophils in autoimmune diseases. The involvement of macrophages in autoimmune disease of the brain is highlighted in a review by X. Fan et al. Although current knowledge is quite limited, the ability of macrophages to polarize in different activation modes and carry out different and often opposing tasks renders them important mediators of the pathogenesis of diseases such as neuromyelitis optica (NMO), myasthenia gravis (MG), and Guillain-Barré syndrome (GBS), and burning questions, such as whether macrophages can be targeted or used as future therapeutic agents, must be further explored.

Concluding this special issue is a detailed review by M. A. Rahat and J. Shakya that draws parallels between the cancerous and autoimmune microenvironments. From the point of view of the immune response, cancer and autoimmune diseases, where the immune system is suppressed or hyperactivated, respectively, are opposites. Nonetheless, many elements in the microenvironments are common in both diseases including hypoxia, angiogenesis, the presence of autoantibodies, and cytokine concentrations. Of course, the critical role that myeloid cells, macrophages, and neutrophils in particular play in these diseases and the detailed understanding of the similarities and differences in the two contexts may eventually lead to novel approaches to immunotherapies.

Collectively, these papers highlight the critical importance of macrophages and neutrophils in the microenvironments of both cancerous and autoimmune diseases. These cells can sense the changing microenvironment and interpret the signals and via complex interactions with other tissue cells and infiltrating immune cells, including the interactions between macrophages and neutrophils themselves, they regulate the progression of the immune response in these diseases [6]. These different interactions could become a focus of research in the field in the coming years. Moreover, although the role that macrophages play in the cancer microenvironment has been extensively studied in the last decade, still some areas deserve more attention, for example, the crosstalk between different microenvironmental factors such as hormones and hypoxia. Neutrophils bear many similarities to macrophages, in their secretome and their killing and proangiogenic abilities and in the way the microenvironment activates them in different and opposing manners. However, this has only recently begun to be unveiled, and more research directed at neutrophil characterization, understanding of their contribution, and deciphering their interactions with different cell populations must be invested. And lastly, the current knowledge and the findings described in this issue point to several new insights into the mechanisms of current and potential therapies and suggest new possible combination therapies that could benefit patients and should be further explored.

*Michal A. Rahat* *Michal A. Rahat* *Seth B. Coffelt* *Seth B. Coffelt* *Zvi Granot* *Zvi Granot* *Munitta Muthana* *Munitta Muthana* *Amedeo Amedei* *Amedeo Amedei*
